# Age of First Walking and Associations with Symptom Severity in Children with Suspected or Diagnosed Autism Spectrum Disorder

**DOI:** 10.1007/s10803-019-04112-y

**Published:** 2019-07-05

**Authors:** Lise Reindal, Terje Nærland, Bernhard Weidle, Stian Lydersen, Ole A. Andreassen, Anne Mari Sund

**Affiliations:** 1 Department of Child and Adolescent Psychiatry, Møre og Romsdal Hospital Trust, Volda Hospital, Pb 113, 6101 Volda, Norway; 2 Regional Centre for Child and Youth Mental Health and Child Welfare, Department of Mental Health, Faculty of Medicine and Health Sciences, Norwegian University of Science and Technology, Trondheim, Norway; 3 NevSom, Department of Rare Disorders and Disabilities, Oslo University Hospital, Oslo, Norway; 4 NORMENT Centre, University of Oslo, Oslo, Norway; 5 Department of Child and Adolescent Psychiatry, St. Olavs Hospital, Trondheim, Norway; 6 Division of Mental Health and Addiction, Oslo University Hospital, Oslo, Norway

**Keywords:** Autism spectrum disorder, Intellectual disability, Motor, Sex differences, Symptom severity, Walking

## Abstract

Age of first walking (AOW) is reported to be later in autism spectrum disorder (ASD) compared with typical development. However, the relationship between AOW and variations in ASD symptoms across different neurodevelopmental disorders is largely unknown. This study investigated AOW and its association with autism symptom severity in a large sample of children (*N *= 490, 23% females) clinically evaluated for suspected ASD, differentiated into ASD (*n *= 376) and non-ASD (*n *= 114) diagnoses. Children with ASD achieved independent walking significantly later than children with non-ASD diagnoses. AOW was significantly associated with ASD symptom severity, and females had a non-significant later AOW. The current findings suggest that in cases with delayed AOW, ASD should be considered as an actual differential diagnosis, perhaps particularly in girls.

Neurodevelopmental disorders (NDD) affect 10–15% of children (Gillberg 2010; Boyle et al. 2011), often presenting with early delay in one or more developmental domains. Autism spectrum disorder (ASD) is a childhood onset NDD characterized by persistent deficits in communication skills and social interaction, as well as restricted, repetitive behavior and interests (RRB) (American Psychiatric Association 2013). Autistic symptoms vary widely both across individuals meeting diagnostic criteria and the general population (Constantino and Todd 2003, 2005; Posserud et al. 2006), and clinicians often face the dilemma of assessing children with autistic symptoms who do not meet the diagnostic criteria for ASD. At present, there is a growing dimensional view of ASD symptoms transcending diagnostic categories (Constantino and Charman 2016; Lord et al. 2018; Ryland et al. 2012). However, studies comparing characteristics of children receiving an ASD diagnosis to those who initially display signs of ASD but do not meet diagnostic criteria are needed.

Although motor performance is not part of the diagnostic criteria for ASD, motor deficits are common (Fournier et al. 2010), have been recognized as an associated feature since the earliest descriptions of the phenotype (Asperger 1944; Kanner 1943), and suggested as a cardinal ASD characteristic (Fournier et al. 2010; Staples et al. 2012; Hilton et al. 2012). Motor signs, such as the attainment of motor milestones, may be more easily and reliably observed than core ASD symptoms. This has led researchers to study early motor delays as a potential pathway for early identification and intervention in ASD. Emerging research has documented differences between ASD and typically developing infants, with higher rates of parent reported concerns about motor development and later attainment of motor skills, including walking among children with ASD (West 2018). Longitudinal data suggest these differences amplify with age (Landa and Garrett-Mayer 2006), and that early motor difficulties may be a risk factor for impaired social communication and cognition, traits that are related to ASD (Leonard et al. 2014). At present, early motor delays are considered to be a prodromal symptom of ASD (Bhat et al. 2012; Harris 2017), although with low specificity, as they are also associated with intellectual (Lemcke et al. 2013) and other developmental disabilities (Zwaigenbaum et al. 2015; Hatakenaka et al. 2016).

Age for onset of independent walking (AOW) is a fundamental and reliable (Hus et al. 2011) parent-reported milestone. Learning to walk is typically achieved around 12 months of age, and AOW at or after 16 months considered an established marker of atypical development (Onis 2006b). The onset of walking is found to support early language development (West et al. 2017; Walle and Campos 2014) and to affect infants’ social interaction (Karasik et al. 2014), suggesting importance not only for later motor skills. Among children with ASD, a deviant pattern of language development following the onset of walking has been reported (West et al. 2017), potentially contributing to the communicative difficulties that characterize ASD. Recently evidence support that delayed AOW may also be an early marker of neurobiological and genetic abnormality in ASD (Bishop et al. 2017; Buja et al. 2018).

Attainment of walking is reported to be later among children with ASD. Estimates vary from 1.1 to 2.5 month delay in mean AOW compared with samples of typically developing children (Ozonoff et al. 2008), children at low risk for ASD (West et al. 2017), and a national birth cohort (Lemcke et al. 2013). Mean AOW has also been reported among different ASD subgroups (Matson et al. 2010; Lemcke et al. 2013; Ozonoff et al. 2008), and for other non-ASD samples with atypical development (Ozonoff et al. 2008; Bishop et al. 2016), intellectual disability (ID) (Lemcke et al. 2013) or language delay (West et al. 2017). Notably, study design, assessment methods, sample sizes and clinical groups used for comparison varied between these studies, hampering comparability and generalization of results. A further methodological limitation has been the lack of normative data regarding AOW. However, this is available in Norway, where the use of both national and regional data (Storvold et al. 2013), as well as comparisons with other countries (Onis 2006b) are considered to increase the external validity and generalizability of the results.

Increased severity of ASD has been related to greater deficits in a multitude of areas. An as yet unanswered question is whether delays in AOW is associated with severity of ASD symptoms across diagnostic categories. Several studies have reported a pattern of slowed motor development across clinical groups (Matson et al. 2010; Ozonoff et al. 2008; Lemcke et al. 2013), where children with ID or general developmental delays show the most delay, followed by ASD subtypes by decreasing severity. Motor skills have also been negatively correlated with symptom severity in autistic children (Hilton et al. 2012) and found to predict autism severity scores in toddlers (MacDonald et al. 2014) and school-age children with ASD (MacDonald et al. 2013), suggesting that motor skills may be related to symptom severity and not just an ASD diagnosis. Because of the high comorbidity of ID in children with ASD, the possible influence of cognitive impairment on early motor delays has been discussed as a limitation of several previous studies. In their sample of 1185 individuals (ASD, *n* = 903; non-ASD, *n *= 282), Bishop et al. (2016) found that lower IQ scores were associated with increased rates of late walking in both ASD and non-ASD groups, but children with low IQ without ASD were more likely to show delayed walking. Among individuals with ASD and nonverbal IQ (NVIQ) above 85, late walking (defined as at or after 16 months) occurred in 13%, against 31% in children with NVIQ less than 70. Female sex was found to heighten risk for delayed walking overall.

ASD is considered to affect males more often than females (Kim et al. 2011). The literature, however, seems biased toward investigating the male profile of ASD (Kirkovski et al. 2013). Given similar levels of ASD symptoms, females appear to require more behavioral/cognitive problems to receive a diagnosis (Dworzynski et al. 2012). Overall, females with ASD are more likely to have neurological abnormalities, less RRB, and worse intellectual and adaptive functioning than males (Lai et al. 2015). Whereas no consistent sex differences in AOW has been observed among typically developing children (Onis 2006a; Jenni et al. 2013; Storvold et al. 2013), there are indications that females with ASD exhibit higher rates of delayed AOW, compared with ASD males (Bishop et al. 2016; Arabameri and Sotoodeh 2015).

Although previous studies have provided useful information regarding AOW as a potential early marker for ASD, whether delays in AOW is associated with severity of ASD symptoms across diagnostic categories remains unclear. We investigated this relationship in a large clinical sample of Norwegian children assessed for suspected ASD by specialist health services, who varied in their severity of symptoms, cognitive abilities, and age at diagnosis. Specifically, we compared AOW, sex, age, NVIQ, and severity of autistic symptoms between children receiving an ASD diagnosis and children not meeting the criteria for diagnosis (non-ASD). Furthermore, we investigated the associations between AOW and symptom severity independent of ASD diagnosis. Finally, we investigated these questions separately for males and females. Available Norwegian population norms for AOW allowed for comparison with typically developing children.

## Methods

### Study Design

This study involved analyses of data collected and processed by August 31, 2017. The study sample is part of BUPgen, an ongoing large multi-site study of neurodevelopmental disorders in Norway, in which children are eligible for enrollment if a suspicion of ASD has been raised by local or specialist health services. Data are collected from two types of sites: (1) child habilitation services and (2) child and adolescent mental health services. These are public specialist health services receiving referrals for assessment of ASD, depending on the presenting symptoms, level of impairment, co-occurrent somatic or psychiatric difficulties, and according to local routines. After written, informed consent to participate, information from patients’ records was extracted by clinicians, following standard procedures.

### Participants

**Fig. 1:**
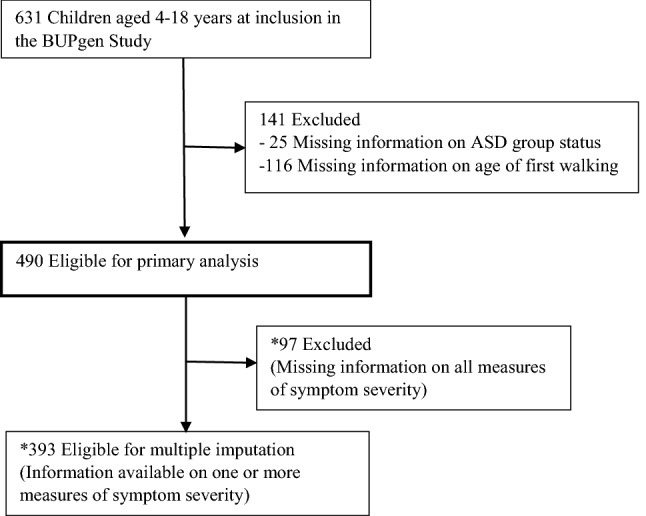
Study sample recruitment flowchart. *Post hoc analyses were performed to assess comparability with the total study sample (*N *= 490)

Participants were eligible if information on age (4–18 years) at inclusion, diagnostic classification as either ASD or non-ASD, and age of first walking (AOW) was available. A total of *N* = 490 children were included, born between the years 1992 and 2012, with a mean (*M*) age at inclusion of 11.1 years (standard deviation (*SD*) = 3.7) (Fig. 1). Data were collected from the clinical evaluation, and included results from present and previous clinical assessments, parent-reported history and supplementary parent-reported measures.

### Diagnoses

All diagnoses were clinical diagnoses, assigned by specialist health services, using the International Statistical Classification of Diseases, 10th Revision (ICD-10) criteria (World Health Organization 1992). Participants were separated into two groups: A total of 376 children with a clinical diagnosis of any ASD according to ICD-10 (F84x) formed the ASD group, and 114 children with suspected autistic symptoms but no clinical ASD diagnosis formed the non-ASD group. The majority of ASD (81.6%, *n* = 307) and non-ASD individuals (56.1%, *n* = 64) had completed the Autism Diagnostic Interview-Revised (ADI-R) (Rutter et al. 2003b), the Autism Diagnostic Observation Schedule (Lord et al. 1999), or both as part of their clinical evaluation. Other NDDs were grouped according to ICD-10 codes: Intellectual disability (ID) (F70–79), Attention-deficit/hyperactivity disorder (F90), Communication disorder (F80), Specific learning disorder (F81 and F83), Motor disorder (F82 and F95), other neurodevelopmental disorder (F88, F89 and F94). The presence of epilepsy or cerebral palsy was also registered.

### Measures

#### Early Motor Impairment

A clinician rated medical history form was filled in for all participants at inclusion, which inquired about age for onset of independent (unaided) walking (in months) (AOW). This form was completed based on available information in the child’s medical record supplemented by parent report, asking parents to retrospectively recall AOW. Comparisons were made with mean AOW from a typically developing population, obtained from (Storvold et al. 2013). They investigated the normal distribution of AOW among Norwegian children (*n* = 47,515), finding a mean AOW of 12.86 (*SD *=1.88) months (95% CI 12.85–12.88). In line with previous reports (Bishop et al. 2016), we defined “late walking” as AOW at or after 16 months.

#### Measures of Autistic Symptoms

The Autism Diagnostic Interview-Revised (ADI-R) (Rutter et al. 2003b; Lord et al. 1994) is a semi-structured caregiver interview consisting of items relevant to the core domains of ASD. The scoring algorithm is based on DSM-IV and ICD-10 criteria, yielding separate scores for social, verbal/nonverbal communication, and RRB domains. The ADI-R has demonstrated high sensitivity and moderate specificity (Lord et al. 1994), with a Cronbach’s α of .69 for the RRB and .95 for the social domain. In our sample, the Cronbach’s α ranged from .49 for the RRB to .82 for the social domain. There are no Norwegian or Scandinavian norms available for the ADI-R, but the inter-rater reliability for single ADI-R algorithm items, behavioral domains totals and agreement for diagnostic classification for the Scandinavian versions is reported to be good (Halvorsen and Helverschou 2017). Following ADI-R conventions as presented by Hus and Lord (2013), and to make scores comparable across participants of different ages and language levels, the ADI-R nonverbal total included totals from the social, nonverbal communication, and RRB domains, leaving 27 items (totals ranged from 9 to 54). Mean age at administration of ADI-R was 9.8 years (*SD *= 3.9).

The Social Communication Questionnaire (SCQ) (Rutter et al. 2003a) comprises 40 yes/no questions, and is completed by caregiver to identify behaviors associated with autism across the lifespan. The SCQ content parallels that of the ADI-R, and excellent agreement (Berument et al. 1999; Bishop and Norbury 2002) and concurrent validity (Rutter et al. 2003a) has been reported. Cronbach’s α for SCQ total lifetime score in the present sample was .89, comparable to previous reports (Rutter et al. 2003a).

The Social Responsiveness Scale (SRS) (Constantino and Gruber 2005) is a 65-item, ordinal-scaled caregiver-report questionnaire examining a child’s ability to engage in reciprocal social interactions. The total score is a valid measure of autistic social impairment, with higher scores indicating greater severity (Constantino and Todd 2003; Constantino et al. 2003). We applied SRS raw total as a dimensional trait variable, for which the Cronbach’s α was .94 in the present sample, comparable to previous reports (Constantino and Gruber 2005).

For simplicity, we use the term “symptom severity” as a proxy for total score on the different measurements of autistic symptoms (ADI-R, SCQ and SRS).

#### Measures of Cognitive Abilities

Cognitive function was assessed using results from previously administered, age-appropriate Wechsler scales: the Wechsler Preschool and Primary Scale of Intelligence (Wechsler 2012; 9.2%), Wechsler Intelligence Scale for Children (Wechsler 2003; 77.8%), Wechsler Abbreviated Scale of Intelligence (Wechsler 1999; 9.2%), and Wechsler Adult Intelligence Scale (Wechsler 2008; 3.7%). These assessments yield standard scores for nonverbal IQ (NVIQ), verbal IQ, and full scale IQ. To minimize the effect of language in measuring cognitive abilities, we used NVIQ as a trait variable, reflecting severity of cognitive impairment. Mean age at assessment of cognitive abilities was 10.2 years (*SD *=3.4).

### Statistical Analyses

We used the independent samples t-test and Pearson’s Chi squared to compare sample characteristics between ASD and non-ASD individuals. AOW was compared with a Norwegian normative sample, for which the mean AOW was 12.86 months (*SD *= 1.88) (Storvold et al. 2013). Cohen’s *d* was computed for effect sizes corresponding to the independent samples t-tests (Cohen 1988). A post hoc analysis of covariance was conducted to compare mean AOW between the two diagnostic groups after controlling for NVIQ. We assessed whether AOW was associated with severity of autistic symptoms by performing linear regression analyses with total scores on the ADI-R, SCQ, and SRS as dependent variables, one at a time. Analyses were carried out unadjusted and adjusted for potential confounders, one at a time and simultaneously. The unique contribution of AOW to predicting the different dependent variables was assessed with squared multiple correlation ($R^{2}$) in unadjusted, and squared semipartial correlation ($sr^{2}$) in adjusted analyses. Potential confounding factors included were cognitive ability (NVIQ) (Levy et al. 2010), prematurity, maternal and paternal age (Lord et al. 2018), which are known risk factors for ASD and may influence AOW. In addition, age at inclusion (years), sex, and ethnicity (both parents of Caucasian ethnicity or not) were included in the adjusted regression models. To explore possible sex differences, group comparisons were repeated for males and females separately. Possible sex differences in the associations between AOW and symptom severity were explored in subsequent regression analyses including an interaction term between sex and AOW, and in separate analyses for each sex.

The number of children who completed the different measures of ASD symptom severity varied from 141 to 335, and 97 children had missing data on all three measures. 151 children did not have available data on NVIQ. Missing values were handled by multiple imputation (MI) on the sample of *n* = 393 individuals with available data on one or more measures of ASD symptom severity, as described in Appendix 1. We report both available case analyses based on the original dataset, and analyses based on MI. Two-sided *p*-values < 5% were regarded as statistically significant. IBM SPSS 25 software was used for statistical analyses, except for comparisons with the normative sample in Stata 15.

## Results

**Fig. 2:**
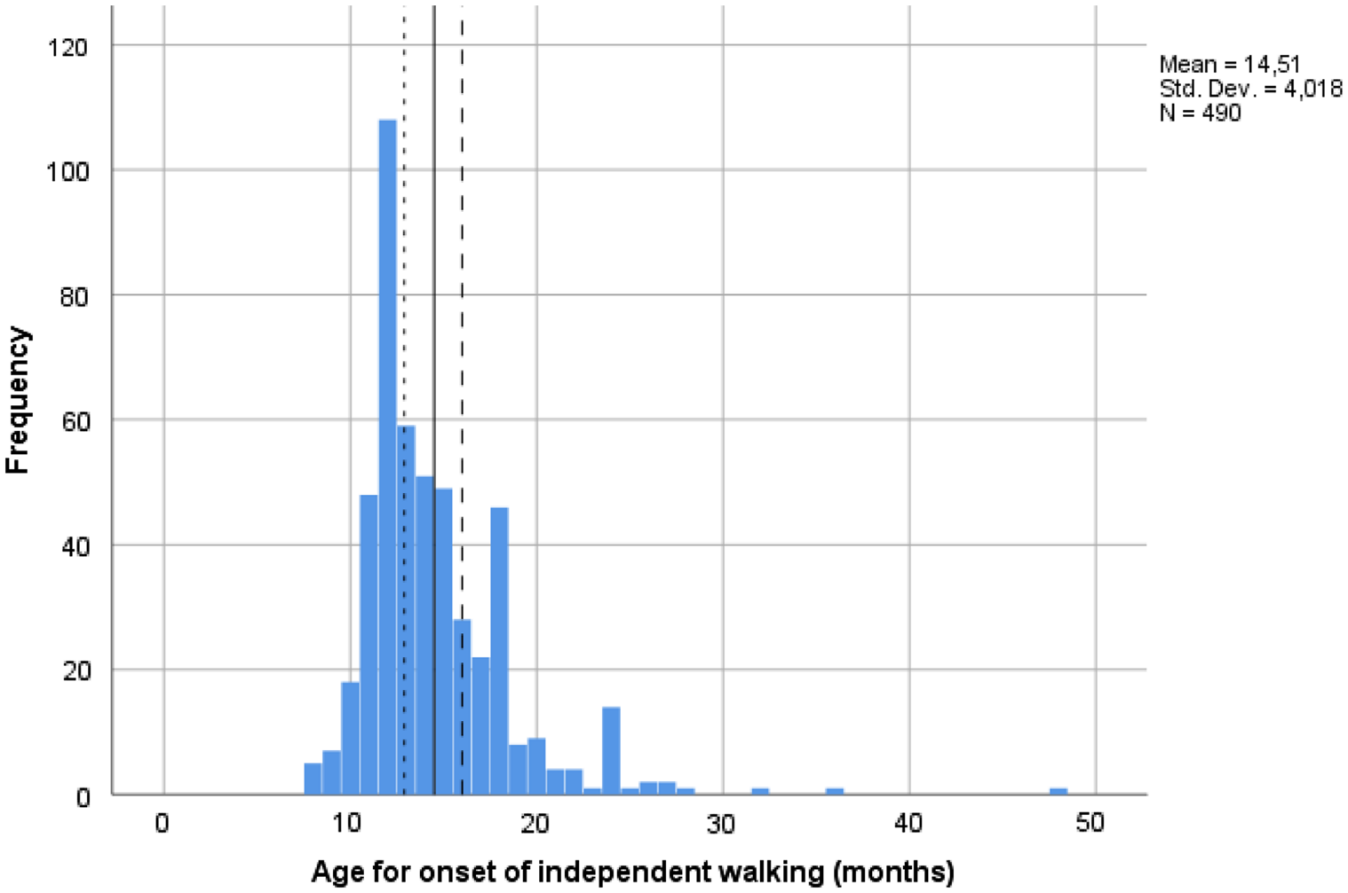
Distribution of age for onset of independent walking (AOW) in the total study sample (*N *= 490). Small and larger stippled lines represent mean AOW among Norwegian children (Storvold et al. 2013) and cutoff for “late walking” (≥ 16 months), respectively; solid line represents mean AOW in the present sample

Among the 376 children with ASD, common subtypes included Asperger syndrome (35.6%), Childhood Autism (29.8%), Pervasive Developmental Disorder Not Otherwise Specified (22.9%), and Atypical Autism (9.6%). Mean age at ASD diagnosis was 9.3 years (*SD *=4.2). The majority of the children in the non-ASD group (92.4%) had one or more NDDs. Having two or more NDDs was more common in the ASD group (192/371, 51.8%) than in the non-ASD group (44/105, 41.9%), although not reaching statistical significance (*p* = .075). All children had achieved independent walking. AOW ranged from eight to 48 months, with a mean of 14.5 months (*SD *= 4.0) (Fig. 2).

### Differences Between ASD and Non-ASD

The main sample (*N* = 490) included 377 males, with a male to female ratio of 3.5:1 in the ASD and 2.9:1 in the non-ASD group (Table 1). Mean NVIQ was in the normal range and did not differ significantly between these groups (*p* = .54). Non-ASD individuals were younger at inclusion, 10.2 years (*SD *= 3.6) versus 11.4 years (*SD *= 3.8) in the ASD group (t (194) = 2.99, *p* = .003). However, mean age at administration of ADI-R did not differ significantly between the diagnostic groups (*p *= .77). ASD individuals had higher scores on all measures of symptom severity compared with non-ASD (p < .001, all) (Table 2).

**Table 1:** Participant characteristics (*N* = 490)

	ASD (*n* = 376)			Non-ASD (*n* = 114)		
	*n*	(%)	Mean (SD)	*n*	(%)	Mean (SD)
Sex						
Male	292	(77.7)		85	(74.6)	
Early motor development						
AOW (months)	376		14.7 (4.3)	114		13.8 (2.9)
“Late walking” (≥ 16 months)	117	(31.1)		28	(24.6)	
Diagnoses						
ASD (F84)	376	(100.0)		0	0	
Former ASD	0	0		6	(5.5)	
Intellectual Disability (F70–79)	49	(15.1)		9	(9.8)	
ADHD (F90)	114	(36.2)		52	(55.9)	
Communication disorder (F80)	12	(3.8)		23	(23.0)	
Specific learning disorder (F81 + F83)	18	(5.7)		22	(23.4)	
Motor disorders (F82 + F95)	35	(11.1)		27	(29.0)	
Epilepsy	25	(6.6)		8	(7.0)	
Cerebral Palsy	1	(.3)		1	(.9)	
Other NDD (F88 + F89 + F94)	4	(1.3)		8	(8.4)	
No of NDDs						
0	0	0		8	(7.6)	
1	179	(48.2)		53	(50.5)	
2–3	182	(49.1)		40	(38.1)	
≥ 4	10	(2.7)		4	(3.8)	
Verbal language	305	(92.7)		100	(100.0)	
Age (years) at inclusion	376		11.4 (3.8)	114		10.2 (3.6)
Age (years) at ASD diagnosis	326		9.3 (4.2)			
Nonverbal IQ	254		102.3 (17.7)	85		100.9 (17.5)
Verbal IQ	258		89.1 (17.8)	86		92.9 (18.0)
Paternal age (years)	213		32.5 (6.3)	69		32.9 (6.5)
Maternal age (years)	231		30.3 (5.0)	73		30.8 (5.5)
Prematurity	50	(14.8)		15	(15.5)	
Ethnicity						
European (Caucasian)	281	(81.2)		98	(89.1)	

*AOW* age for onset of independent walking, *ASD* autism spectrum disorder, *ADHD* attention-deficit/hyperactivity disorder, *NDD* neurodevelopmental disorder. Data are expressed as *n* (%) or mean (SD). The denominator for the reported proportions in this table excludes those with missing data: 151 participants for nonverbal IQ; 146 for verbal IQ; 61 for language level; 56 for prematurity; 34 for ethnicity; 208 for paternal age; 186 for maternal age; 14 for number of NDDs, and 52 ASD cases for age at diagnosis. IQ was obtained from various age-appropriate standardized tests

**Table 2:** Mean score on measures of autistic symptom severity and comparisons between diagnostic groups

	ASD			Non-ASD			95% CI for the difference
	*n*	Range	Mean (SD)	*n*	Range	Mean (SD)	
Available case analysis (*n* ≤ 490)							
SCQ total	143	2–34	16.2 (7.2)	72	0–24	8.1 (5.6)	(6.4 to 9.9)***
ADI-R nonverbal total	118	0–45	22.0 (8.9)	23	0–38	11.9 (9.8)	(6.0 to 14.2)***
SRS total	247	7–168	86.8 (28.6)	88	19–130	64.5 (25.2)	(15.5 to 29.1)***
Multiple-imputation (*n* = 393)							
SCQ total	296		15.0 (6.4)	97		9.4 (5.9)	(4.2 to 7.0)***
ADI-R nonverbal total	296		22.3 (16.5)	97		12.5 (22.0)	(4.8 to 14.9)***
SRS total	296		85.9 (26.4)	97		66.1 (24.5)	(14.1 to 25.6)***

Measures of autistic symptom severity: *SCQ* Social Communication Questionnaire, *ADI-R* Autism Diagnostic Interview-Revised, *SRS* Social Responsiveness Scale. Results based on available case analyses of the main sample and multiple imputation of *n* = 393 participants with data on at least one measure of autistic symptom severity. *SD* standard deviation, *CI* confidence interval, *p*  *p*-value

*p < .05; **p < .01; ***p < .001 for independent samples t-tests

**Fig. 3:**
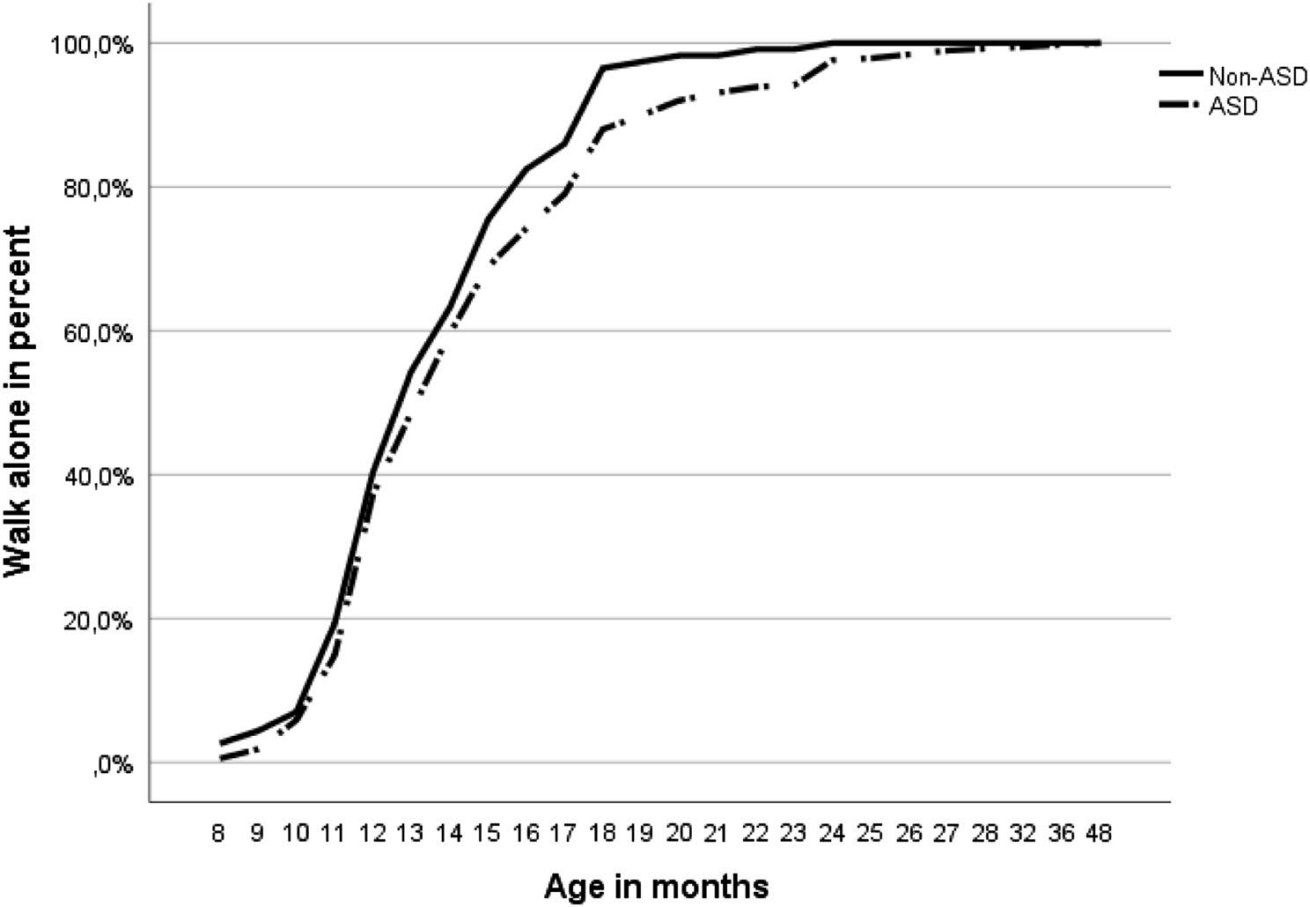
Cumulative proportion of children having attained independent walking at increasing ages (per parent report), based on ASD group status (*N *= 490). Information is included through age 48 months. ASD = autism spectrum disorder, Non-ASD = assessed for autistic symptoms, but without ASD diagnosis

Figure 3 illustrates the proportion for having attained independent walking at increasing ages in both groups. A widening gap appears from age 12–13 months, reaching a maximum at 18–19 months, at which time more non-ASD individuals had attained walking. Applying a “cut off” for AOW at 16 months, the extent of “late walkers” was found to be somewhat higher in the ASD group (117/376, 31%) compared with the non-ASD group (28/114, 25%), although not statistically significant (*p* = .22).

Mean AOW among ASD children, however, was later compared with non-ASD, 14.7 (*SD *= 4.3) versus 13.8 (*SD *= 2.9) months, respectively (*t* (278) = 2.80, *p *= .005; *d *= 0.34). Compared with the normative sample (Storvold et al. 2013) (stippled line in Fig. 2), mean AOW was later among ASD (mean difference 1.9 months, *t* (376) = 8.51, *p *< .001; *d *= 0.55), as well as non-ASD individuals (mean difference 0.9 months, *t* (113) = 3.33, *p *< .001; *d *= 0.38). When adjusting for NVIQ in a post hoc analysis of covariance, mean AOW remained significantly later in the ASD group compared with non-ASD in the imputed dataset (mean difference 0.9 months, *p* = .04). In available case analysis (n = 339 due to missing information on NVIQ) mean AOW did not differ significantly between the ASD and non-ASD group: 14.0 (*SD* = 3.0) versus 13.7 (*SD *= 2.9) months, respectively (*p* = .33). This finding can be explained by the result that the 151 children with missing data on NVIQ had a later AOW (mean of 15.8, *SD* = 5.5 months) and 80% were diagnosed with ASD, as further discussed in the Appendix.

A proportion of children in the non-ASD group (29% vs 11% in the ASD group, *p *< .001) were diagnosed with motor disorder, all of which were Tic disorders. Four children were also diagnosed with F82 (i.e. they had both F82 and F95). Within the non-ASD group, mean AOW among children diagnosed with motor disorder was earlier, however not significant (*p *= .35), compared with those not diagnosed with a motor disorder (n = 66); 13.4 months (*SD* = 2.6) versus 14.0 (*SD *= 2.8), respectively.

### AOW and Autistic Symptom Severity

Delays in AOW was associated with increasing symptom severity (Table 3). The strongest association was found between AOW and ADI-R, with AOW explaining 5.4% of the variance in ADI-R nonverbal total score ($R^{2}$ = .054, *p* = .005) in unadjusted analyses. After adjustment with potentially confounding variables, the association remained significant, with AOW explaining 7.0% of the total variance ($sr^{2}$ = 0.070, *p* = .02). The association between AOW and SCQ lost its significance after adjustment in the available case analysis, but remained significant (*p* = .02) after adjustment in the imputed dataset. Otherwise, there were no major changes in the significance of parameter estimates between the original and imputed data. The association between AOW and SRS was non-significant and therefore not subject to further analyses.

**Table 3:** Linear regression with measures of autistic symptom severity as dependent variables and AOW as primary covariate

Available case analysis (n ≤ 490)	SRS total				SCQ total				ADI-R nonverbal total			
	*n*	B	CI	*p*	*n*	B	CI	*p*	*n*	B	CI	*p*
*Unadjusted*												
AOW (months)	335	.72	(− .15 to 1.59)	.10	215	.55	(.24 to .86)	.001	141	.69	(.21 to 1.18)	.005
*Adjusted separately for*												
Sex (female)					215	.57	(.26 to .89)	< .001	141	.66	(.18 to 1.15)	.007
Age (years)					215	.56	(.24 to .87)	.001	141	.69	(.21 to 1.18)	.005
Nonverbal IQ					178	.15	(− .24 to .53)	.44	123	.48	(− .10 to 1.06)	.11
Prematurity					199	.61	(.29 to .92)	< .001	129	.81	(.30 to, 1.33)	.002
Ethnicity (non-Caucasian)					209	.52	(.20 to .84)	.001	137	.72	(.24 to 1.21)	.004
Paternal age (years)					130	.47	(.05 to .89)	.03	98	.95	(.39 to 1.50)	.001
Maternal age (years)					146	.46	(.08 to .83)	.02	102	.85	(.30 to 1.40)	.003
*Adjusted for all*	151	− .37	(− 1.82 to 1.09)	.62	103	.23	(− .25 to .70)	.35	75	.88	(.14 to 1.62)	.02

Multiple imputation (n = 393)	SRS total				SCQ total				ADI-R nonverbal total			
	*n*	B	CI	*p*	*n*	B	CI	*p*	*n*	B	CI	*p*
*Unadjusted*												
AOW (months)	393	.61	(− .13 to 1.35)	.11	393	.25	(.07 to. 43)	.007	393	.93	(.39 to 1.46)	.001
*Adjusted separately for*												
Sex (female)					393	.26	(.07 to .44)	.006	393	.94	(.41 to 1.47)	.001
Age (years)					393	.25	(.07 to .44)	.007	393	.93	(.40 to 1.46)	.001
Nonverbal IQ					393	.23	(.04 to .42)	.02	393	.84	(.29 to 1.39)	.003
Prematurity					393	.24	(.05 to .42)	.01	393	.94	(.41 to 1.47)	.001
Ethnicity (non-Caucasian)					393	.25	(.07 to .43)	.008	393	.92	(.39 to 1.46)	.001
Paternal age (years)					393	.25	(.07 to .44)	.007	393	.92	(.39 to 1.45)	.001
Maternal age (years)					393	.25	(.07 to .43)	.007	393	.93	(.39 to 1.46)	.001
*Adjusted for all*	393	.48	(− .30 to 1.27)	.23	393	.24	(.05 to .44)	.02	393	.83	(.29 to 1.37)	.003

*AOW* age for onset of independent walking. Dependent variables: *SCQ* Social Communication Questionnaire, *ADI-R* Autism Diagnostic Interview-Revised, *SR*S Social Responsiveness Scale. Results based on available case analysis of the main sample and multiple-imputation analysis of *n* = 393 participants with data on at least one dependent variable. *B* unstandardized regression coefficient, *CI* 95% confidence interval, *p p*-value

### Sex Differences

AOW, NVIQ, and symptom scores, as well as between-group comparisons within each sex, are presented in Table 4. Among all children with suspected ASD, AOW was later among females (*M *= 15.0, *SD *= 4.5) than males (*M *= 14.4, *SD *= 3.9), but at a non-significant level (*p* = .16). Mean AOW was later among males with ASD compared with males in the non-ASD group-14.6 (*SD *= 4.2) versus 13.4 (*SD *= 2.4) months, respectively (*t* (238) = 3.50, *p* = .001; *d *= 0.53)-but did not differ between groups in females. Females with ASD exhibited the latest AOW among all groups (*M *= 15.1, *SD *= 4.7), with a mean difference of 2.2 months (*t* (83) = 4.37, *p *< .001; *d *= 0.62), compared with the normative sample, in which no sex difference was found (Storvold et al. 2013). Adjusting for NVIQ in separate post hoc analyses for each sex did not alter the significance of observed group differences in mean AOW.

**Table 4:** AOW, nonverbal IQ, and symptom scores in ASD versus non-ASD, as well as between-groups comparisons

		ASD females		Non-ASD females	95% CI for the difference		ASD males		Non-ASD males	95% CI for the difference
	*n*	Mean (SD)	*n*	Mean (SD)		*n*	Mean (SD)	*n*	Mean (SD)	
Available case analysis (*n* ≤ 490)										
AOW (mo)	84	15.1 (4.7)	29	14.9 (3.8)	(− 1.7 to 2.1)	292	14.6 (4.2)	85	13.4 (2.4)	(0.6 to 2.0) **
Nonverbal IQ	53	99.4 (13.9)	23	98.2 (14.7)	(− 5.9 to 8.2)	201	103.0 (18.6)	62	101.9 (18.4)	(− 4.2 to 6.4)
SCQ total	32	15.6 (7.5)	17	7.0 (6.3)	(4.3 to 12.9) ***	111	16.4 (7.2)	55	8.4 (5.3)	(6.0 to 10.0) ***
ADI-R nonverbal total	28	20.7 (9.7)	8	7.4 (7.1)	(5.8 to 20.8) **	90	22.4 (8.7)	15	14.3 (10.4)	(3.2 to 13.1) **
SRS total	51	93.5 (26.4)	21	61.0 (26.5)	(18.9 to 46.2) ***	196	85.1 (29.0)	67	65.7 (24.8)	(11.7 to 27.2) ***
Multiple-imputation analysis (*n* = 393)										
AOW (mo)	63	14.7 (4.6)	24	15.0 (3.7)	(− 2.5 to 1.7)	233	14.6 (3.6)	73	13.3 (2.3)	(0.6 to 2.0) ***
Nonverbal IQ	63	99.6 (17.8)	24	98.6 (15.2)	(− 6.6 to 8.6)	233	102.2 (25.9)	73	103.0 (19.9)	(− 6.5 to 5.0)
SCQ total	63	14.5 (7.2)	24	8.4 (6.9)	(2.8 to 9.4) ***	233	15.1 (6.1)	73	9.7 (5.6)	(3.9 to 6.9) ***
ADI-R nonverbal total	63	21.5 (19.7)	24	9.2 (22.1)	(1.5 to 23.1) *	233	22.5 (16.0)	73	13.5 (20.9)	(3.5 to 14.5) **
SRS total	63	91.7 (24.2)	24	63.7 (25.9)	(16.3 to 39.5) ***	233	84.3 (26.8)	73	66.8 (24.2)	(10.6 to 24.4) ***

*ASD* autism spectrum disorder, *AOW* age for onset of independent walking. Measures of autistic symptom severity: *SCQ* Social Communication Questionnaire, *ADI-R* Autism Diagnostic Interview-Revised, *SRS* Social Responsiveness Scale. Results based on available case analysis of the main sample and multiple imputation of *n* = 393 participants with data on at least one measure of symptom severity. *SD* standard deviation, *CI* confidence interval, *p p*-value

*p < .05; **p < .01; ***p < .001 for independent samples t-tests

The interactions between sex and AOW were not significant in analyses predicting symptom severity. In separate analyses for each sex, associations remained significant among males but not females (Table 5). Looking at the regression coefficients, however, they were of the same magnitude in females and males in both datasets in unadjusted analyses, indicating that for each month increase in AOW, the burden of autistic symptoms as measured by ADI-R increased approximately as much in each sex.

**Table 5:** Linear regression among females and males with measures of symptom severity as dependent variables and AOW as the primary covariate

Available case analysis (*n* ≤ 490)	Females								Males							
	SCQ total				ADI-R nonverbal total				SCQ total				ADI-R nonverbal total			
AOW (mo)	*n*	B	CI	*p*	*n*	B	CI	*p*	*n*	B	CI	*p*	*n*	B	CI	*p*
Unadjusted	49	.31	(− .27 to .88)	.29	36	.64	(− .59 to 1.87)	.30	166	.72	(.34 to 1.11)	< .001	105	.67	(.15 to 1.19)	.01
Adjusted for all*	23	− .52	(− 1.44 to .39)	.24	22	1.15	(− .91 to 3.21)	.25	80	.51	(− .10 to 1.12)	.10	53	.37	(− .487 to 1.22)	.39

Multiple imputation (*n* = 393)	SCQ total				ADI-R nonverbal total				SCQ total				ADI-R nonverbal total			
AOW (mo)	*n*	B	CI	*p*	*n*	B	CI	*p*	*n*	B	CI	*p*	*n*	B	CI	*p*
Unadjusted	87	.21	(− .16 to .57)	.27	87	.87	(− .30 to 2.04)	.15	306	.28	(.07 to .49)	.008	306	.98	(.41 to 1.54)	.001
Adjusted for all*	87	.16	(− .22 to .54)	.40	87	1.02	(− .17 to 2.21)	.09	306	.31	(.09 to .54)	.007	306	.78	(.18 to 1.37)	.01

*AOW* age for onset of independent walking. Dependent variables: *SCQ* Social Communication Questionnaire, *ADI-R* Autism Diagnostic Interview-Revised. Results based on available case analysis of the main sample, and multiple imputation of *n* = 393 participants with data on at least one dependent variable. *B* unstandardized regression coefficient, *CI* 95% confidence interval, *p p*-value

*Adjusted for the covariates: age, nonverbal IQ, prematurity, ethnicity, maternal and paternal age

## Discussion

In this study of AOW in a large sample of Norwegian children assessed for suspected ASD by specialist health services, we found that mean AOW was later among children with ASD compared to their typically developing peers, consistent with previous reports (Ozonoff et al. 2008; Lemcke et al. 2013; West et al. 2017). AOW was associated with severity of core autistic symptoms, even after adjustment for potential confounders. Whereas AOW was significantly later in males with ASD compared with non-ASD diagnosis, females with autistic symptoms seem to have a liability toward later AOW, regardless of ASD diagnosis.

To our knowledge, this is the first study of AOW among children evaluated for suspected ASD, and directly aimed at investigating associations with symptom severity and possible sex differences. Applying a dimensional approach, we found that among children who displayed signs of ASD without meeting the criteria for diagnosis (non-ASD), AOW was significantly later compared with norms for typically developing children, but to a less extent than in children with ASD. Consistent with our results, Lane et al. (2012) found that in a small sample (n = 30) of young children referred for possible ASD, those who received an ASD diagnosis tended to have greater delays in fine and gross motor domains, although not statistical significant, compared with children not diagnosed as ASD.

In the present study, symptom severity was higher in the ASD group compared with non-ASD, but with some overlap on all measures. Such overlap may be unavoidable, reflecting genetic relationships between ASD and other developmental disorders (Lichtenstein et al. 2010; Lundstrom et al. 2011). Our findings support the concept of autistic symptoms as quantitative traits transcending diagnostic categories (Frazier et al. 2015). Further, a pattern emerged, where AOW seems to represent a continuum along which children with ASD show the most delay, followed by those with fewer autistic symptoms. This is in line with previous findings indicating that the more severe the autistic symptoms, the greater the likelihood of co-occurring conditions (Lundstrom et al. 2011) and functional difficulties (Skuse et al. 2009), including motor difficulties (Matson et al. 2010; Green et al. 2009; Hilton et al. 2012; MacDonald et al. 2013, 2014). Regarding AOW, a similar pattern of observed delay has been reported in retrospective (Ozonoff et al. 2008) as well as prospective (Lemcke et al. 2013) studies. The latter, a Danish national birth cohort study, reported increasing delay in AOW across different conditions, with the longest delay among children with ID and not ASD, followed by childhood autism and then any ASD diagnosis, including childhood autism. Extending previous studies, we included children with autistic symptoms without an ASD diagnosis. The lack of a control group was mitigated by using normative AOW data from the same population (Storvold et al. 2013). While significant differences in mean AOW between groups and compared to norms was found, most children in both groups did attain walking within 16 months. The proportion of children characterized as “late walking” (i.e., AOW at or after 16 months) was smaller but considerable; 31% of the ASD and 25% of the non-ASD group. Our findings contrast somewhat with a recent study by Bishop et al. (2016), in which 22% of 903 children with ASD were “late walking”, with mean AOW 14.00 (4.73) months.

Children with ASD are reported to have high frequencies of one or more co-occurring neurodevelopmental, psychiatric, and possibly causative medical diagnoses (Levy et al. 2010; Lord et al. 2018). Other diagnoses or symptoms may be present before all the symptoms of ASD are evident. In a prospective study of 30 children referred for early motor delays or abnormalities, including delayed walking (Hatakenaka et al. 2016), the majority were found to have at least one NDD. Thirteen children were later diagnosed with ASD, of which 92% had two or more NDDs. Also in the present sample NDDs were common; 52% in the ASD and 42% in the non-ASD group had two or more NDDs. Moreno-De-Luca et al. (2013) have argued that “neurodevelopmental disorders should be thought of as different patterns of symptoms or impairments of a common underlying neurodevelopmental continuum”. As such, the possibility that the observed common co-occurrence of NDDs in the present sample may represent a common etiology or underlying issues affecting also the motor domain, should be considered. In the present sample, 29% in the non-ASD and 11% in the ASD group were diagnosed with ‘motor disorder’. This category comprised ICD-10 diagnoses F82 (Specific developmental disorder of motor function) and F95 [Tic disorders, including Tourette’s disorder (F95.2)], see Table 1, the majority of which were Tic disorders. The inclusion of motor disorder had a negligible effect on the main results.

Although it is possible to make a diagnosis of ASD before 24 months age in some cases, the majority of children with ASD in northern Europe are diagnosed by early school age (Lord et al. 2018). In the present sample, mean age at ASD diagnosis was 9.3 years. Our results are consistent with Suren et al. (2012) who used nationwide Norwegian register data and found that the proportions with ASD from 2008 to 2010 increased by age and was 0.7% in 11-year-olds. This suggests that ASD is often not diagnosed until late childhood or early adolescence in Norway. Later diagnoses are reported to occur in the context of co-occurring problems and other factors (e.g. female sex, more advanced language) that might have either exacerbated or masked the ASD (Lord et al. 2018). The present study included children from both child habilitation services and child and adolescent mental health services evaluated for suspected ASD. This enabled the inclusion of individuals with a broad range of autistic symptoms and cognitive abilities. We consider this to strengthen the representativity of our results for the broader population of individuals assessed for suspected ASD in the health care system.

Taken together, the relatively high number of females, individuals with ASD subtypes without language delay (36% had Asperger syndrome) and the high proportion with co-occurring NDDs may have contributed to the relatively late age at ASD diagnosis in our sample. In terms of cognitive functioning, individuals with ASD display a wide range of abilities, from severe ID to superior intelligence, with prevalence rates for ID in different studies between 15 and 65% (Lord et al. 2018). In our sample, 15.1% of ASD and 9.8% of non-ASD individuals were diagnosed with ID, further indicating a more ‘high functioning’ sample. Applying a dimensional approach, we included children with a broad range of autistic symptoms despite having other co-occurring disorders. In our sample, 27 children with ASD and seven in the non-ASD group had known genetic conditions, some of which may have contributed to later AOW in both groups, and later AOW compared to other ASD samples with more strict exclusion criteria. Further, Norwegian children are *on average* older at AOW, compared with other countries (Storvold et al. 2013; Onis 2006b).

Our finding of mean AOW at 14.7 months in the ASD group is later compared with some earlier reports (Lemcke et al. 2013; Bishop et al. 2016). The magnitude of delay, however—children with ASD walking *on average* almost 2 months later compared with typically developing children—is comparable to previous studies (Lemcke et al. 2013; Ozonoff et al. 2008; West et al. 2017). This highlights the need to assess AOW in relation to autistic symptoms. The strongest association between AOW and symptom severity was found for ADI-R, with AOW making a unique contribution in explaining ADI-R total score. This held after adjusting for potential confounders. The association between AOW and SCQ was lost following adjustment in available case analyses, but remained significant after adjustment in the MI sample, which is considered less biased and to strengthen our results. A weaker association between AOW and SCQ may be reasonable, however, given that SCQ is a short parent-report questionnaire allowing only yes/no answers, whereas ADI-R is a semi-structured interview requiring trained examiners, which may perform better in eliciting parental concerns and capturing current and historical ASD symptoms. Further, the SCQ is found to be more similar to the ADI-R total score in differentiating ASD from non-ASD in the older (8-10, > 11) than younger age groups (Corsello et al. 2007). Contrary to our finding that AOW was associated with symptom severity, as measured by the ADI-R and SCQ, and previous reports of correlations between SRS and motor skills (Hilton et al. 2007, 2012), we found no significant association between AOW and SRS. This may indicate that SRS captures other aspects of social impairment that are not as strongly associated with AOW, compared with measures of core autistic symptoms.

When assessing relationships between ASD symptoms and other behavioral or neurobiological variables, taking into account phenotypic characteristics, such as age, IQ or co-occurring difficulties is important. ASD symptom measures such as the SRS and ADI-R are reported to capture more than symptoms of ASD, with elevating scores potentially reflecting impairments in dimensions other than the core characteristics of ASD (Havdahl et al. 2016). The possibility that early motor delays are more general signs of compromised neurocognitive development, rather than specific to ASD, has also been discussed (Bolton et al. 2012; Ozonoff et al. 2008). Of the covariates included in the regression model in the present sample, NVIQ was making the strongest contribution to attenuating the relation between AOW and severity of core ASD symptoms. Significant associations remained, however, as did the difference in mean AOW between the ASD and non-ASD groups after adjusting for NVIQ. Thus, in our sample AOW was related to ASD symptom severity, even after adjusting for NVIQ. In order to examine whether AOW predicts ASD symptom severity over and above general motor ability, results from broader measures of motor functioning would have been useful. Unfortunately, such a measure was not included in the present study.

Because of potential typical sex differences, it is important to compare how males and females with ASD differ from typically developing males and females (Lai et al. 2015). The WHO Multicentre Growth Reference Study (MGRS) found no significant, consistent sex differences in motor milestone achievement ages among typically developing children (Onis 2006a). However, “girls in the MGRS tended to achieve milestones at earlier ages than did boys” (p. 71). Contrary to this, but in line with previous reports (Bishop et al. 2016; Arabameri and Sotoodeh 2015), we found that females with autistic symptoms (regardless of ASD diagnosis) are more liable to *delayed* walking compared with males. Findings from screening-negative infants later diagnosed with ASD (Oien et al. 2018) have highlighted the discrepancy between categorical criteria for ASD and developmental signs of an emerging or subthreshold autism phenotype (Oien et al. 2018). Specifically, girls had less advanced early gross motor skills compared with boys. Along with a recent report from a large population study that autistic social traits in females tend to increase towards adolescence (Mandy et al. 2018), these results may indicate a different phenotype or emerging pattern of symptoms in females with ASD.

Strengths of our study include the sample size and inclusion of individuals with a broad range of autistic symptoms and cognitive abilities. In addition, we used validated instruments, and the nature of data collection allowed adjustment for covariates and potential confounding factors. Study limitations are the retrospective nature of some of the information collected in the study, varying measures of autistic symptoms, and missing data (se Appendix for further discussion). We used clinical diagnoses obtained from different clinics, which may have introduced variation. Misclassification (in both directions) is possible, but not very probable for ASD and the non-ASD disorders, and is unlikely to be related to AOW assessment. ASD diagnoses assigned by Norwegian specialist health services have previously shown high overall validity (Suren et al. 2012). Further, the relatively high number of females in our sample may indicate that referral and ascertainment bias leading to under recognition of ASD in females (Lai et al. 2015) was low. For some analyses regarding sex differences our sample may have been underpowered. Otherwise, we do not consider type I errors to be likely. Nevertheless, these findings should be replicated in independent samples. Finally, the lack of control group was overcome by using normative AOW data from the Norwegian study by Storvold et al. (2013). In that study, information on AOW was collected by parent report when children were 18 months of age, whereas we used retrospective information on AOW collected at inclusion (age from 4 to 18 years), introducing the possibility of recall bias. The quality of information about developmental milestones from caregivers has been examined by Hus et al. (2011), who found AOW to be one of the most reliable parent report measures (Hus et al. 2011). Although the precision of information regarding AOW may have varied, it is unlikely to have systematically biased our results. Further, the pattern and magnitude of delay observed is in accordance with results from previous studies.

Children with ASD share common features with children with other developmental delays, which may contribute to difficulties of accurate diagnosis. Although delayed onset of walking is not unique to ASD, the present study supports previous reports that it occurs commonly in ASD, and further demonstrate associations with severity of symptoms in other diagnostic criterion domains that characterize ASD. Recognizing that autistic symptoms may be difficult to interpret at an early age, assessing early motor delays and specifically AOW may have the potential to improve earlier identification of some cases with ASD, and perhaps particularly in females. Considering the possibility of ASD in infants with motor delays may not only enhance the potential for earlier diagnosis, but also improve the chance of targeting and addressing these delays in treatment programs and facilitate better prognostic outcomes.

## Conclusion

Our results showing later onset of independent walking among children with ASD compared to children who display symptoms of ASD without meeting diagnostic criteria, highlight the importance of assessing AOW in relation to autistic symptoms. The current findings suggest that AOW may constitute a continuum parallel to the continuum of autistic symptoms, with potential sex effects. In cases with delayed AOW, ASD should be considered as an actual differential diagnosis, taking particular notice of females. The underlying mechanisms and clinical implications should be investigated in prospective studies.

## Appendix: Missing Data, Multiple Imputation and Sensitivity Analyses

The number of children with available data on the different measures of symptom severity varied. For ADI-R, subscales that sum to the ADI-R nonverbal total (social, nonverbal communication, and RRB domains) were complete for 141 subjects, 329 subjects were missing all subscales, and 20 were missing one or more subscales. For SCQ, subscales were complete for 210 subjects, five subjects had a total value available but no subscales, and 275 were missing all data. For SRS, subscales were complete for 335 subjects, one was missing one or more subscales, and 154 were missing all data. SRS raw scores were based on caregiver report. For three participants, a teacher was the informant. Clinician-reported data were missing for 151 participants for nonverbal IQ, 146 for verbal IQ, 61 for language level, 56 for prematurity, 34 for ethnicity, 208 for paternal age, 186 for maternal age, 74 for ID diagnose, and 52 ASD cases for age at diagnosis. Information on age at administration of ADI-R was available for 188 children.

Primary demographic and clinical characteristics of the total study cohort (*N* = 490) were compared with *n* = 97 individuals with missing data on all measures of symptom severity, and *n* = 393 individuals with available data on one or more measures (Tables 6, 7). Levels of missing data tended to be comparable for males and females but more common among those who where younger, nonverbal, and/or with lower cognitive performance. More members of the sample with data missing for all measures of symptom severity had an ASD diagnosis, and mean AOW was later. The same pattern was observed among the 151 children with missing data on NVIQ, where mean AOW was 15.8 (*SD *= 5.5), and 80% were diagnosed with ASD. Hence, data were not missing completely at random (MCAR), but possibly missing at random (MAR). As such, estimates of symptom scores in the available case analyses may be biased toward higher functioning (in the ASD group), making group comparisons and estimates of associations more conservative.

**Table 6:** Participant characteristics across samples

	*n *= 490 Main sample			*n *= 97 Missing data on symptom severity			*n* = 393 Multiple imputation sample		
	*n*	(%)	Mean (SD)	*n*	(%)	Mean (SD)	*n*	(%)	Mean (SD)
Sex									
Male	377	(76.9)		71	(73.2)		306	(77.9)	
AOW (months)	490		14.5 (4.0)	97		15.1 (5.2)	393		14.4 (3.7)
Age (years) at inclusion	490		11.1 (3.7)	97		9.3 (4.1)	393		11.5 (3.5)
Verbal language	405	(94.4)		30	(76.9)		375	(96.2)	
Nonverbal IQ	339		101.9 (17.7)	14		94.4 (16.8)	325		102.2 (17.6)
Verbal IQ	344		90.1 (17.9)	15		82.2 (14.2)	329		90.4 (18.0)
Paternal age (years)	282		32.6 (6.3)	28		32.7 (6.6)	254		32.6 (6.3)
Prematurity	65	(15.0)		18	(23.1)		47	(13.2)	
Ethnicity									
European (Caucasian)	379	(83.1)		56	(60.2)		323	(89.0)	

Data are expressed as *n* (%) or mean (SD). The denominator for proportions reported in this table excludes those with missing data. Nonverbal IQ was obtained from various age-appropriate standardized tests

*AOW* age for onset of independent walking, *ASD* autism spectrum disorder

**Table 7:** Characteristics of participants with missing data on symptom severity (*n *= 97)

	ASD (*n* = 80)			Non-ASD (*n* = 17)		
	*n*	(%)	Mean (SD)	*n*	(%)	Mean (SD)
Sex						
Male	59	(73.8)		12	(70.6)	
AOW (months)	80		15.3 (5.5)	17		14.1 (3.4)
Age (years) at inclusion	80		9.5 (4.1)	17		7.9 (3.5)
Verbal language	27	(75.0)		3	(100.0)	
Nonverbal IQ	12		96.9 (14.5)	2		79.5 (29.0)
Verbal IQ	13		85.1 (13.0)	2		63.5 (3.5)
Paternal age (years)	25		32.8 (5.8)	3		31.7 (13.3)
Prematurity	16	(23.9)		2	(18.2)	
Ethnicity						
European (Caucasian)	45	(59.2)		11	(64.7)	

Data are expressed as *n* (%) or mean (SD). The denominator for proportions reported in this table excludes those with missing data. Nonverbal IQ was obtained from various age-appropriate standardized tests

*AOW* age for onset of independent walking, *ASD* autism spectrum disorder

Missing data were handled using multiple imputation (MI), creating m = 100 imputed data sets as recommended by Van Buuren (2018). Available case analysis is unbiased only if data are MCAR, while multiple imputation analysis is unbiased under the less restrictive MAR assumption. The wealth of data collected through BUPgen allowed multiple imputation to include auxiliary variables associated with missingness, increasing the plausibility that the MAR assumption is a realistic approximation of reality. All variables used in subsequent analyses were included in the imputation model, with the following modifications: For ADI-R the four subscales (social, verbal communication, nonverbal communication, and RRB domains) were used in imputation. For SCQ, we included the total and the four subscales that summed to the total, without constraints. For SRS, we included the five subscales and the total. The variable ADI-R nonverbal total was computed after imputation. In addition, language level, ASD diagnosis and verbal IQ were also included in the imputation model. To accommodate interactions with sex, we imputed files separately for males and females, and merged the imputed files, as described by Van Buuren (2018, pp. 175, 176). We did not restrict the imputed values to the scale range, as recommended by Rodwell et al. (2014).

The variable language level was dichotomized as verbal or nonverbal based on information at inclusion. Individuals were considered to be nonverbal if: (1) they had received ADOS Module 1 as part of their clinical evaluation, designed for children who are nonverbal or using single words; (2) they were reported to not combine words and use sentences (question 1 in the SCQ and/or question 30 in the ADI-R); or (3) clinician-reported information at inclusion otherwise indicated that they were nonverbal. Information on language level was used as a categorical indicator of expressive language to help explain data missingness in the imputation model, but was not included as a covariate in further analyses. The variables verbal IQ and nonverbal IQ were obtained from previously administered age-appropriate measures of cognitive ability.

Due to missing information on AOW, 116 children were not eligible for the present sample. Primary demographic and clinical characteristics for these children, however, was comparable to those of the total study cohort (*N* = 490). To assess the potential impact of outliers on mean AOW of the total study sample, as well as estimates of group differences and associations with symptom severity, we used z-scores, finding 5 observations (1.0%) with z-scores > 2.58, and 15 observations (3.1%) with z-scores > 1.96. Thus, the number of potential outliers did not deviate much from what expected within a normal distribution. Further, main analyses were repeated after removing first the most extreme, thereafter the two most extreme values (AOW $\ge$ 36 months), resulting in a modest attenuation of the results, not affecting the statistical significance of the difference between groups.
